# Voltage-Gated Sodium Channel Dysfunction in Epilepsy: Zebrafish Models for Therapeutics

**DOI:** 10.3390/biomedicines13092078

**Published:** 2025-08-26

**Authors:** Angela Gyamfi, Priyadharshini Manikandan, William A. Cisneros, Theodore R. Cummins, James A. Marrs

**Affiliations:** Department of Biology, Indiana University Indianapolis, Indianapolis, IN 46202, USA; angyamfi@iu.edu (A.G.); pmanikan@iu.edu (P.M.); wcisnero@iu.edu (W.A.C.); trcummin@iu.edu (T.R.C.)

**Keywords:** epilepsy, zebrafish, voltage-gated sodium channels, seizures, channelopathies, ion channels, *SCN1A*, *SCN2A*, *SCN3A*, *SCN8A*

## Abstract

Voltage-gated sodium channels (VGSCs) play pivotal roles in cellular function, particularly in the regulation of electrical signaling. Structural defects in these channels cause deleterious effects in a myriad of cell types, leading to various diseases, like epilepsy, cardiac arrythmias, kidney disease, and certain cancers. Over the past decade, significant efforts have been geared toward developing drugs that target the pore domains of these channels, called pore-blocking agents. This approach has seen several setbacks, commonly due to the lack of isoform-specific binding. Alternative targeting strategies are being used to reduce or eliminate the side effects of pore-blocking agents. Transgenic mouse models have proven useful in such studies, and subtype-selective inhibitors were developed. The zebrafish model system was also used to explore neurological, cardiovascular, and metabolic diseases caused by voltage-gated sodium channel dysfunction. Here, we delve into the growing literature on the structure and function of voltage-gated sodium channels, their role in epilepsy and its comorbidities, and the advancement in the use of zebrafish as a model system to explore these channels as therapeutic targets.

## 1. Voltage-Gated Ion Channels

Ion channels are assemblies of integral proteins that span the plasma membrane and form pores that act as highly selective gates regulating the flow of specific ions in or out of the cell in response to environmental cues [1]. They are generally classified into voltage-gated, ligand-gated, and mechanosensitive ion channels based on their activation mechanisms or gating properties [2]. There are subclasses of channels based on their ion-selective permeability, including sodium (Na^+^), potassium (K^+^), chloride (Cl^−^), and calcium (Ca^2+^) [3]. Voltage-gated sodium channels (VGSCs) are transmembrane proteins that are selectively permeable to Na^+^ ions [4]. They are highly sensitive to changes in cell membrane potential and are dispersed across excitable cells like neurons [3,4]. When the membrane potential of a cell reaches a specific threshold, VGSCs open and allow a rapid influx of Na^+^ ions, causing depolarization [4]. This depolarization is quickly followed by repolarization, a process mediated by voltage-gated potassium channels [5]. These potassium channels open shortly after sodium channels, allowing the efflux of K^+^ ions, which restores the membrane potential to its resting state [5]. This process generates action potentials essential for transmitting electric signals [6]. Structural studies conducted on VGSCs revealed that they are composed of 24 transmembrane segments arranged into four domains, which include the voltage sensor region and pore-forming segments [7]. These domains differ in their functional and structural properties but altogether allow the channel to independently assume an open (activated), inactivated (refractory period), or resting state, regulating the influx of Na^+^ ions and generating the inward current [7,8]. VGSCs play a pivotal role in regulating metabolic, neurological, and cardiac processes, as these processes rely heavily on the generation of electrical signals. These channels are therefore essential drivers of excitability in cells such as neurons, muscle cells, endocrine pituitary cells, and non-excitable cells such as astrocytes, Schwann cells, T cells, and macrophages. Defects in VGSCs can lead to channelopathies, resulting in a variety of disorders, such as epilepsy, and studying their functions in model systems like zebrafish provides a platform to develop potential treatments.

## 2. VGSC Subunits

### 2.1. α Subunit Structure

**Figure 1:**
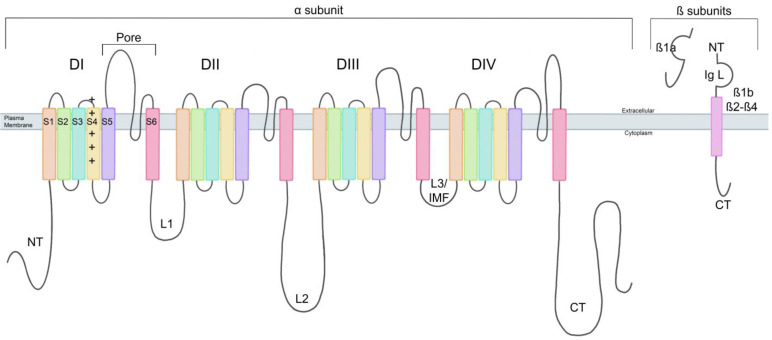
VGSC α and β subunit structure. The α subunit is made up of four domains (DI–DIV), each composed of six transmembrane segments (S1–S6). Within each domain, the S4 segment functions as the voltage sensory component, while the S5 and S6 are pore-forming subunits, contributing to forming the channel pore. The domains are connected by intracellular loops (L1–3), and L3 is also known as the IFM structure. The N-terminus (NT) is cytoplasmic and located before S1 of DI, while the C-terminus (CT) is also cytoplasmic, located after S6 of DIV. There are five β subunits (β1a, β1b, β2–β4). All five have an NT, CT, and immunoglobin loop (Ig L). β1b and β2–β4 have a transmembrane domain, while β1a does not, as it is a secreted molecule.

**Figure 2:**
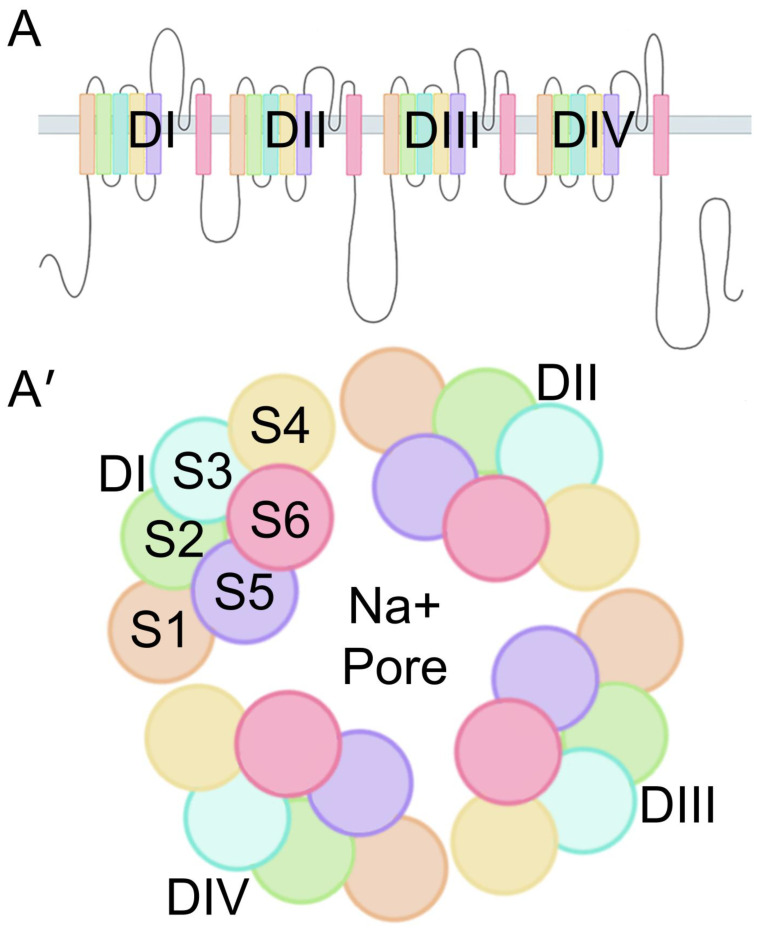
VGSC pore formation. (**A**) Linear model through plasma membrane of VGSC α subunit. (**A′**) *En face* view of α subunit formation of Na^+^ pore on plasma membrane. DI–DIV arranged clockwise, showing the 6 α-helixes (segment1–6 or S1–S6) with all S5 and S6 subunits ordered in the center, creating the Na^+^ pore.

VGSCs are composed of a pore-forming α subunit and usually two associated β subunits. The α subunit is functionally dominant, but co-expression of the β subunits may be required to exhibit normal Na^+^ channel electrophysiological function. In humans, there are nine homologous proteins (Nav 1.1–Nav 1.9) that are encoded by genes *SCN1A–SCN5A* and *SCN8A–SCN11A*, respectively [9,10]. VGSCs are distributed across the central and peripheral nervous system (CNS and PNS); Nav 1.1, Nav 1.2, Nav1.3, and Nav 1.6 isoforms are found across the CNS, and Nav 1.7, Nav 1.8, and Nav 1.9 isoforms are found across the PNS [11]. Alternative splicing and transcriptional modifications produce various isoforms of each Na^+^ channel, altering sensitivity, kinetics, and distribution. The single polypeptide chain that makes up the α subunit folds into four related but nonidentical transmembrane domains (DI–DIV), three intracellular loops (L1–L3), and the N- and C-terminus (NT and CT) domains (Figure 1). The four repeating transmembrane domains assemble, creating the ion-selective (Na^+^) pore in the center (Figure 2A′) [12]. Each transmembrane domain comprises six hydrophobic α helix transmembrane segments (S1–S6). S4 on each domain is highly sensitive to changes in the membrane potential, making it the voltage sensory component (Figure 1) [13]. L1 and L2 are long loops that connect DI to DII and DII to DIII, respectively, whereas L3 is a short loop connecting DIII to DIV and contains the IFM structure, which has three hydrophobic amino acid residues (Isoleucine–Phenylalanine–Methionine). The IFM structure facilitates intracellular signaling, leading to rapid inactivation of the VGSCs (Figure 1 and Figure 2) [4].

### 2.2. α Subunit Function

**Figure 3:**
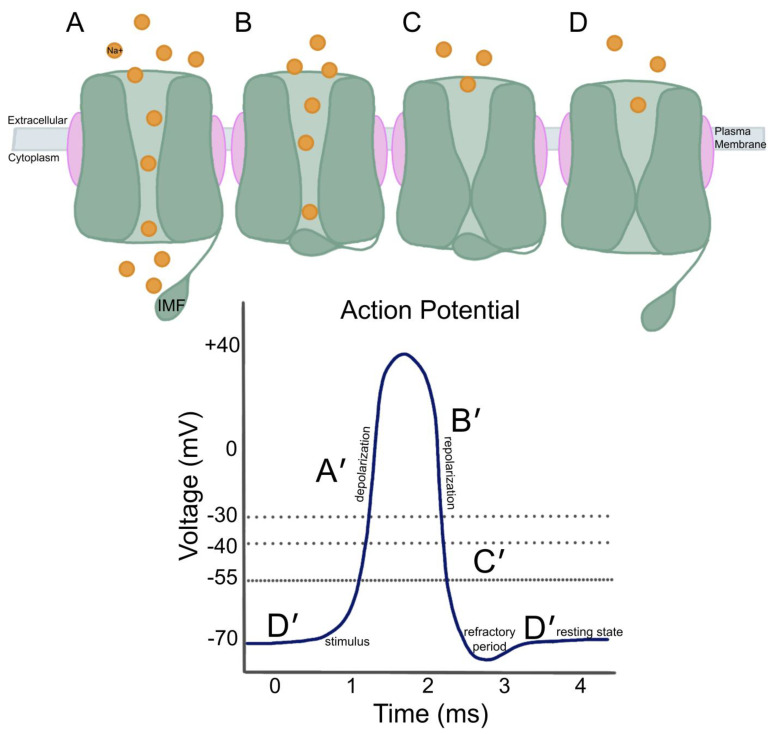
VGSC gating model and function of an action potential. (**A**–**D**) VGSC conformational changes over time to an active and inactive gate. The α subunit contains an Na^+^ pore in the center, a voltage-gated activation gate towards the bottom (shown by grey arrows), and the intracellular IFM/time-dependent inactivation gate. β subunits are located on either side of the α subunit. (**A′**–**D′**) Depiction of an action potential measured by membrane potential voltage in millivolts (mV) over time in milliseconds (ms). The resting membrane potential of a neuron is around −70 mV (**D′**). When a sufficient stimulus is applied, initiating an action potential, an influx of Na^+^ ions must raise the membrane potential to −55 mV, triggering depolarization (**A′**) up to around +40 mV. This is followed by repolarization (**B′**,**C′**) and hyperpolarization, where membrane potential drops below −70 mV, caused by an efflux of K^+^ ions, and a refractory period occurs before the neuron returns to its resting state (**D′**). (**A**,**A′**) Open/activated gate during depolarization events, allowing for flow of Na^+^ into cell. (**B**,**B′**) Fast inactivation of gate by IFM occluding pore, halting Na^+^ influx, during repolarization from more positive membrane potentials down to around −30 mV; the inactivation gate remains open. (**C**,**C′**) Slow close of inactivation gate while IFM still plugs the pore, allowing for the cell to repolarize at more negative potentials around −40 mV to below. (**D**,**D′**) Closed/inactivated gate, IFM releases from plugging the pore, membrane potential is back to a resting state at −70 mV.

VGSCs propagate action potentials by undergoing voltage-dependent activation, rapid inactivation, and Na^+^ selectivity (Figure 3) [4,13]. Nav channels control cellular depolarization through a complex gating mechanism (Figure 3A–D) [13,14]. Two gates operate in the α subunit: a voltage-gated activation gate and a time-dependent inactivation gate [13]. The pore module is made up of S5, S6, and a hairpin-like pore loop connecting the two segments (Figure 1) [15]. The activation gate is composed of the intracellular bottom halves of the pore-lining S6 segments, and the inactivation gate is formed by L3, which contains the IFM [14,15]. VGSCs create mechano-electrical feedback where voltage-sensitive proteins are conformationally responsive to voltage potential changes across the membrane [14]. At resting membrane potential, the pore is in a closed formation and requires depolarization to be activated and opened (Figure 3D,D′) [16]. Opening of the intracellular gate, located in the pore, is triggered by voltage-dependent movement of at least three of the four voltage sensors, allowing for an influx of Na^+^, causing depolarization during an action potential (Figure 3A,A′) [13,14,16,17]. The exact mechanosensitive mechanism allowing for the opening of the pore is unknown, as it is only in the open confirmation for 1–2 milliseconds, and there is debate about different models of opening [14,15]. The “mechanosensitive opening” (MSO) model and the “mechanosensitive activation” (MSA) model are two models that seek to explain mechanosensitivity in the activation of a bacterial VGSC. In the MSO model, opening of the pore occurs through mechanical force on the pore itself, whereas in the MSA model, mechanical stress activates a mechanosensitive protein releasing signaling molecules, which then bind VGSCs and change gating properties [14]. Strege, Cowan, Alcaino, Mazzone, Ahern, Milescu, Farrugia, and Beyder [14] propose that the activation gate follows the MSO model, specifically a swinging door model, where in-plane expansion caused by mechanical stress on the lipid bilayer alters force on the bilayer (force-from-lipid model), leading to expansion or contraction, which causes pore gating and ultimately bends the S6 segments in the middle like door hinges, with non-covalent bonds latching them [14,15].

A time-dependent fast inactivation occurs to stop Na^+^ flow after a few milliseconds, even with continuous stimulation (Figure 3B) [13,15]. The inactivation gate is occluded as the IFM rapidly swings inwards from the cytosol into its receptor, a hydrophobic pocket between the intracellular end of S6 and the S4–S5 linker, following the hinged-lid or ball-and-chain mechanism [15,16]. Immediate plugging of the inactivation gate halts the influx of Na^+^, although the activation gate remains open (Figure 3B). This occurs through open-state fast inactivation of the gate during repolarization from more positive membrane potentials down to around −30 mV (Figure 3B′) [12,15,16,18,19,20]. This permits the cell to repolarize to a more stable state, which enables the gate to slowly close, assuming its closed position through the movement of S4 narrowing the pore (Figure 3C), which occurs through closed-state fast inactivation as the IMF is closing the pore during repolarization from more negative potentials of around −40 mV to more negative potentials (Figure 3C′), functionally closing the activation gate [12,15,16,18,19,20]. Lastly, the IFM releases into a relaxed position as the VGSC is closed, and the membrane potential goes back to a resting state at −70 mV (Figure 3D,D′) [12,15,16,18,19,20].

### 2.3. β Subunit Structure

β subunits were originally characterized as auxiliary to the pore-forming α subunit but are now known to be multifunctional in excitable and non-excitable cells that can function with and without the α subunit. There are five types of associated β subunits (β1A, β1B, β2–β4) (Figure 1). β1 has two variants, β1A and β1B, which are encoded by *SCN1B*, and β2–β4 are encoded by *SCN2B*–*SCN4B*, respectively [4,21]. β subunits are part of the immunoglobulin (Ig) superfamily of cell adhesion molecules (CAMs) due to their large extracellular V-set Ig loop [21,22]. Most β subunits consist of three parts, an NT extracellular Ig loop, a transmembrane domain, and a CT cytoplasmic domain; β1a does not contain a transmembrane domain, making it a secreted molecule (Figure 1) [4,22,23]. Variants of β1 and β3 are linked to the α subunit through non-covalent bonds with their NT and CT and are highly similar in sequence, and β2 and β4 are linked through covalent bonds through their NT and are also highly homologous to one another [4,24]. The α subunit typically associates with two β subunits, one non-covalently bound (β1 or β3) and one covalently bound (β2 or β4) [4].

### 2.4. β Subunit Function

β subunits regulate α subunit excitability through gating kinetics, voltage dependence, and expression of the Na^+^ channel [4]. β4 has been proposed to have a unique ability to promote resurgent currents, which are unusual currents that can promote high-frequency action potential firing and are originally found in Purkinje neurons in the cerebellum. β4 may comprise an alternative inactivation particle on its CT, which can rapidly inactivate an open Na^+^ pore and prevent binding of the fast inactivation gate. Resurgent currents evoked by unbinding of an alternative inactivation particle during repolarization (e.g., during the falling phase of an action potential) can quickly convert the channel to an open state without conventional recovery [12,25,26], generating repetitive action potential firing. There is limited work on the specific purpose of resurgent currents, but they are essential in coordination of motor activities [27]. In a diseased state, Na^+^ channels may be unable to properly maintain dynamics between the depolarized or repolarized state, leading to improper electrophysiological function.

## 3. Sodium Channelopathies

Channelopathies are a group of ion channel disorders caused by disturbances in the ion channel or regulatory proteins, with sodium channelopathies specified to disrupt the Na^+^ channel [8]. They may arise in several different cell types or organ systems, as ion channels are expressed in both excitable and non-excitable cells [8,28,29]. A wide range of neurological, cardiac, and muscle disorders arise from changes in membrane excitability caused by ion channel mutations in excitable cells [30]. Sometimes referred to as “disorders of membrane excitability,” these disorders include but are not limited to epilepsy, migraine, chronic pain, cardiac arrythmia, myotonia, congenital long QT syndrome, hyperkalemic periodic paralysis, and pulmonary and systemic hypertension [4,7,28,31]. Mutations causing channelopathies can be inherited, following an autosomal pattern where both dominant and recessive mutations can exert an effect, but there are numerous instances of de novo mutations that can be devastating and are not transmitted genetically [32,33]. Acquired factors, such as autoimmune disorders and toxin exposure, can also cause channelopathies [8,28]. Channelopathies are difficult to precisely classify due to considerable heterogeneity, phenotypic variability, and several comorbidities, such as seizures, paralysis, myotonia, neurodevelopmental delay, or arrhythmia [8,34]. Ambiguous diagnoses complicate appropriate therapeutics, causing many patients to undergo nonspecific, inconsistent, and marginally effective treatments [34]. Sodium channelopathies occur by disruptions in either the α or β subunit of the Na^+^ channel, disrupting normal Na^+^ channel dynamics and producing improper electrophysiological function. Mutations are difficult to classify because a single amino acid substitution could alter multiple aspects of Na^+^ channel function [35]. Mutations in four evolutionary and functionally similar α subunit genes, *SCN1A*, *SCN2A*, *SCN3A*, and *SCN8A*, lead to neurological diseases, including various types of epilepsy [35,36].

## 4. Epilepsy: Incidence, Prevalence, and Types

Epilepsy is one of the most common neurological disorders, second only to stroke, affecting about 1% of the world’s population [37]. According to the International League Against Epilepsy (ILAE), epilepsy is multifaceted, emanating from the brain and characterized by abnormal brain activity and spontaneous recurrent seizures [38]. Not all seizure types fall within the classification of epilepsy. Exceptions include febrile seizures caused by high fever, seizures resulting from head injury, and non-epileptic seizures; despite their outward similarity to epileptic seizures, they do not involve electrical discharge [37,39]. This variation contributes to misdiagnosis; as such, the ILAE advises that an epileptic seizure must be unprovoked and occur at least 24 h apart [37].

At the molecular level, epilepsy is characterized by hyperexcitability and hypersynchrony of neuronal firing [40,41]. Normally, the resting membrane potential of a neuron is −60 to −70 mV and requires an influx of Na^+^ to reach a threshold of −55 mV to trigger the all-or-nothing depolarization to around +40 mV to fire an action potential. The action potential transmits a signal and also leads to subsequent hyperpolarization past −70 mV caused by an efflux of K+ and a refractory period until resting membrane potential is regained [42]. In epileptic states, this normal orchestration of membrane potential goes awry. Mutations in the sodium channel may lead to a decrease in threshold for action potential, where action potentials are triggered at lower voltage [41]. When multiple neurons are hyperexcitable, this creates a state of hypersynchrony, leading to an epileptic episode [41]. While some mutations partially block Na^+^ channel function, many, such as those found in Dravet syndrome, are truncating and can lead to complete loss of channel function [41,43].

Epilepsy can be attributed to genetic mutations, environmental influences, and secondary brain insults such as trauma, tumors, autoimmune disorders, stroke, and neurogenerative diseases [40,41,44]. Seizures are classified based on parts of the cerebral cortex involved and seizure appearance. Mostly, seizures begin in the temporal lobe and are categorized as focal or generalized during onset [45]. Focal seizures involve a small, localized network of neurons, and generalized seizures involve a bilateral distribution of the involved neurons [37,46]. Patients may be conscious of the ongoing seizure and undergo automatisms, involuntary movements or behaviors. Focal seizures can spread to both cerebral hemispheres, leading to tonic–clonic movements (stiffening and twitching) [47]. Generalized epilepsies have a diverse range of spectrums; however, they all mainly stem from genetic predisposition or mutations, and as such, are referred to as genetic generalized epilepsies (GGEs) or idiopathic generalized epilepsies (IGEs) [48]. Different types of generalized seizures include myoclonic, absence, atonic, tonic, clonic, and tonic–clonic seizures [48,49]. All but myoclonic seizures involve a loss of or alteration in consciousness [46]. Genetic epilepsies have a genetic component or proven heritable traits as risk factors [50]. Many studies have detected genes that contribute to genetic epilepsies. These studies are often first tested in monogenic and dizygotic twins, ruling out environmental causes as risk factors and identifying several monogenic (where a mutation in a single gene causes a phenotype) and polygenic syndromes [48]. GGEs comprise about 4% of all epilepsies [48,51]. The most common types of GGEs are childhood and juvenile absence seizures, juvenile myoclonic epilepsies, and generalized tonic–clonic seizure on awakening [52].

Epilepsy treatment can be fraught with difficulties. It is noteworthy that there are some types of epilepsy with unknown causes. The different symptoms associated with epileptic seizures depend on the location in the brain and the age and sex of the individual, making epilepsy a highly heterogenous condition with variable long-term prognostic patterns [53]. While some cases report a 50% self-remission rate, epilepsy is often difficult to treat and manage [37]. Before epilepsy treatment can be prescribed, it must be determined whether the events were in fact seizures and whether they were unprovoked [46]. Some current treatments for epileptic seizures include anti-seizure medications (ASMs), gene therapy, and surgical excision of brain tissue. Common ASMs are ion channel inhibitors or modulators of neurotransmitters such as GABA (gamma-aminobutyric acid) and glutamate [54]. GABA is an inhibitory neurotransmitter that is released from GABAergic nerve terminals, acting on both GABAA, a ligand-gated ion channel, and GABAB, a G-protein-coupled potassium channel, which are receptors resulting in fast or slow hyperpolarization, respectively [55]. Glutamate is an excitatory neurotransmitter that is released from glutamatergic nerve terminals to AMPA (α-amino-3-hydroxy-5-methyl-4-isoxazolepropionic acid) and kainite receptors, which are permeable to Na^+^ ions and are involved in fast depolarization, and NMDA (N-methyl-D-aspartate) receptors, which are permeable to Na^+^ and Ca^+2^ ions due to a voltage-dependent blockade that is only activated by prolonged depolarization such as epileptiform discharges [55]. Most ASMs work to modulate voltage-gated ion channels, enhance GABA-mediated inhibition, inhibit glutamate receptor synaptic excitation, or direct modulation of synaptic release [55]. Treatment for epilepsy typically involves the use of broad-spectrum antiseizure drugs; however, these often have deleterious effects, and oftentimes response becomes refractory [56]. Sodium channel blockers like carbamazepine, phenytoin, oxcarbazepine, lacosamide, lamotrigine, and topiramate tend to be effective against seizures resulting from gain-of-function mutations but can worsen seizures resulting from loss-of-function mutations [57,58]. Although there are about 30 different ASMs available as epilepsy treatment options, the variable, multifaceted nature of the disease necessitates personalized treatment options.

## 5. Zebrafish as a Model for Epilepsy

Zebrafish have gained popularity as a model system for human diseases due to their high sequence homology with human disease-related genes (84%) [4,59,60]. They have been successfully used to model cardiovascular, metabolic, developmental, motor, and neurological disorders [61]. While their nervous system is simpler (for example, lack of cortical circuits), they nevertheless share key brain regions and structures with mammals, such as the somatosensory network and the subcortical system [62], making them effective for simulating disease processes [63]. Though rodent models were historically used for epilepsy [64] before the first zebrafish seizure model by Baraban et al. [65], rodents are expensive to maintain and often require invasive techniques for experimental studies, which raises ethical concerns (Table 1) [66]. Generally, zebrafish are ideal for large-scale genetic and therapeutic drug screening [67]. Their small size, fast growth rate, and simpler anatomy make them cost-effective and relatively easier to maintain and genetically modify (Table 1). By utilizing advanced genetic manipulation tools such as CRISPR-Cas9, ENU (N-ethyl-N-nitrosourea) mutagenesis, morpholino oligonucleotide (MO) gene knockdown, transcription activator-like effector nucleases (TALENs), zinc finger nucleases (ZFNs), and synthetic capped mRNA injections, researchers are able to efficiently generate mutations or stable transgenic lines, or alter gene expression (Table 1) [66]. In addition, chemically inducing seizures using pentylenetetrazol (PTZ) (GABA receptor antagonist) or kainic acid in zebrafish provides useful models for studying neuronal activity and seizure dynamics [66,68]. Behavior assays including locomotion tracking, thigmotaxis, and prey capture behavior analyses have been developed, through translation to humans requires careful interpretation [69]. An example is, seizures in zebrafish, which are often characterized by hyperactivity followed by loss of posture. Importantly, zebrafish embryos and larvae up to 5 dpf are not considered capable of nociception, which raises fewer ethical concerns compared to rodents (Table 1) [70,71]. Some limitations of the zebrafish, like gene paralogs and developmental biology differences, must be considered. Also, preclinical research using relevant models, like rodents or pluripotent stem cell-derived organoids, would be necessary to validate findings in the zebrafish model.

Electroencephalogram (EEG) recordings, a form of electrophysiology and a standard method of measuring epilepsy in clinical settings, are also applicable to zebrafish studies [78]. There are three forms of electrophysiology, based on the placement of the electrodes relative to the neuron being measured: extracellular, intracellular, and patch-clamp recordings [79]. In extracellular recordings, the electrode is placed just outside the neuron of interest to detect action potentials [79]. In intracellular recordings, the electrode is inserted directly into the neuron to measure membrane potential [79]. In patch-clamp recordings, the electrode is placed such that it makes contact with the neuronal membrane, forming a tight seal [79]. Extracellular field potential recordings localized to a specific brain region (local field recordings) are often used in zebrafish [73,74,79,80]. Here, a microelectrode is placed superficially on the brain of a live zebrafish embedded in low-melt agarose, and measurement is carried out in current clamp mode (where the amplifier maintains a constant current) and the extracellular summed potentials from multiple neurons around the electrode are recorded [74,80]. This technique often presents several advantages, such as the ability to capture direct electrical activity from an intact behaving animal, less invasiveness, and prolonged monitoring of brain activity either from a single or multiple larvae [74,81]. Although standard patch-clamp and intracellular recordings are more challenging to conduct in zebrafish models due to the smaller cell size, zebrafish protocols such as in vivo whole-cell patch-clamp and extracellular multichannel microelectrode arrays are well established (Table 1). The use of genetic calcium reporters such as GCaMP also enables visualization of neuronal hyperactivity, making real-time optical monitoring of brain activity possible [82,83]. These altogether make zebrafish a practical model for studying seizure behavior and epilepsy from the embryonic stages to adulthood [67].

### Zebrafish SCNA Genes

There are currently nine identified *SCNA* VGSC α subunit genes in humans (*SCN1A*–*SCN5A*, *SCN8A–SCN11A*) (Table 2) [84]. Mutations in these genes are associated with various syndromes, such as Dravet syndrome, generalized epilepsies with febrile seizure plus (GEFS+), early- and late-onset infantile seizures, sudden unexpected death in epilepsy (SUDEP), developmental and epileptic encephalopathy, and other neurodevelopmental disorders without seizures, like autism [85,86]. The *SCNA* genes all have corresponding orthologs in the zebrafish, which allow for the assessment of functional consequences through gene knockouts or knockdown studies (Table 2). Zebrafish *scna* genes are made up of four sets of paralog genes (resulting from genome duplication), namely *scn1laa* and *scn1lab*, *scn4aa* and *scn4ab*, *scn5laa* and *scn5lab*, and *scn8aa* and *scn8ab* (Table 2) [87]. They also have conserved orthologs for all four mammalian β subunit genes: *zbeta1*, *zbeta2*, *zbeta3*, *zbeta4.1* and *zbeta4.2*), which exhibit extensive alternative splicing [88]. These genes are differentially expressed in excitable tissues and have been shown to promote the activity of the alpha subunits through co-expression studies of the zebrafish beta subunits with the zebrafish Nav 1.5 in a Chinese hamster ovary expression system [88]. Unlike many other duplicated zebrafish genes, which diverge from their original expression patterns or lose their functions, the zebrafish *scna* paralogs retain their function and are all expressed as early as 10–120 hours postfertilization (hpf) during embryonic development [87]. These eight *scna* genes have high sequence similarity across their transmembrane domains, with the duplicates having much higher sequence similarity in their coding regions [87]. Through sequence comparison, it has been shown that the most divergent regions between these duplicated genes lie in the regions that code for the linkers between the membrane-spanning repeats (DI–DIV), the large CT, and the 3′-untranslated regions (UTRs) [87]. Among all eight *scna* genes, *scn8aa* and *scn8ab* are the most identical (88%), and the only divergent sequence lies at the 3′-untranslated region (UTR). These two paralogs are the least identical when comparing all eight *scna* genes [87]. As reported in previous studies, zebrafish *scn1laa* and *scn1lab* are homologs to the human *SCN1A* gene; they also have been found to be phylogenetically similar to *SCN2A*, *SCN3A*, and *SCN9A*, with *scn4aa* and *scn4ab* being the most identical to *SCN4A*, *scn5Laa* and *scn5Lab* being the most identical to *SCN5A* (and *SCN10A*, *SCN11A*), and *scn8aa* and *scn8ab* being the most identical to *SCN8A* (Table 2).

Ion channel disorders like epilepsy are also studied in mammalian cells transfected with cDNA of the disease gene of interest [69]. The most commonly used cell lines are human embryonic kidney cells (HEKs) and Chinese hamster ovary cells (CHOs) [69]. They are of epithelial origin and have only small endogenous currents, which permits patch-clamp recordings of recombinant channels, but they do not possess a neuronal background, limiting their utility for functional studies [69]. Xenopus oocytes have also been used in studies assessing channel physiology and ion transport. They are great exogenous expression systems; however, they are a non-neuronal expression system, and as such, the cells exogenously express ion channels and can lack auxiliary proteins needed for normal function and regulation [69]. These limits affect the ability of these exogenous systems to accurately represent the full dynamics of channelopathies. Altogether, these model systems can show similar results to endogenous channels but also produce altered results due to the exogenous system environment [69]. Functional understanding can be derived by analyzing differences and similarities in these exogenous systems, and this understanding will help us identify optimal systems and approaches, accelerating drug discovery and the translation to therapy.

## 6. Key *SCNA* Genes Implicated in Epilepsy

Increases in genetic testing have prompted numerous reports identifying four VGSC genes of significant clinical importance in epilepsy: *SCN1A*, *SCN2A*, *SCN3A*, and *SCN8A* [92,93]. Mutations (gain of function/loss of function) in these genes frequently result in diverse epilepsy phenotypes, presenting varying clinical symptoms and responsiveness to treatments [57,86,94]. Like most genes, the spatiotemporal regulation of *SCNA*’s gene expression plays critical roles in determining the resulting phenotype [93]. Mutations in different *SCNA* genes can also produce similar phenotypes, making it difficult to pinpoint the specific gene causing a particular phenotype [57,93]. From a variant analysis study conducted by Ref. [57], across all mammalian SCNAs, variants that occur in the pore–loop S5–S6 regions, which form the large ion filter, typically have loss-of-function effects, whereas those occurring in inactivation regions typically have gain-of-function effects [57]. This is based on in silico analysis and may differ in vivo. The regions identified tend to be conserved between humans and mice, but recent reports show that these regions may not be fully conserved in zebrafish [57]. Notably, great advances have been made towards using zebrafish to screen for ASMs and gene knockout studies to model different seizure-inducing *scna* mutations. Novak, Taylor, Pineda, Lasda, Wright, and Ribera [87] report that all eight zebrafish *scna* genes are expressed during the embryonic and/or the larval stages of development and therefore can effectively be used to model early childhood epilepsies.

### 6.1. SCN1A

The human *SCN1A* gene encodes the alpha subunit pore-forming domain of the VGSC subtype 1 (Nav 1.1). This is the primary sodium channel found in GABAergic interneurons [95]. GABAergic interneurons are predominantly found in the hippocampus, making up about 10–15% of the total neuronal cell population [95]. They play essential roles in the brain during early development and throughout growth. Specifically, they induce fast inactivation of neuronal membranes and contribute to fast depolarization during the initiation of action potentials [96]. The *SCN1A* gene has about ~1300 recorded point mutations arising from the coding region, making it a clinically relevant epilepsy gene [97]. These *SCN1A* mutations are associated with genetic epilepsies, including Dravet syndrome (DS)/severe myoclonic epilepsy in infancy (SMEI) and genetic epilepsy with febrile seizures plus (GEFS+) [95].

#### 6.1.1. Dravet Syndrome

Dravet syndrome (DS) is a severe developmental and epileptic encephalopathy, with most cases caused by heterozygous loss-of-function mutations in the *SCN1A* gene, resulting in haploinsufficiency [98,99]. *SCN1A* haploinsufficiency leads to reduced levels of GABA (inhibitory neurotransmitter in the CNS) [100]. GABA-level reduction causes reduced inhibition of neuronal excitability [100]. Hence, individuals become more susceptible to seizures. Individuals (mostly children under 2) with DS often develop drug-resistant seizures; show developmental delays, cognitive deficits, sleep disorders, and behavioral disorders; and are at risk for sudden unexpected death in epilepsy (SUDEP) [98,101,102,103]. Studies in the field have consistently shown that loss-of-function mutations in the *SCN1A* gene are the primary cause of DS, accounting for about 80% of all cases. In addition, there are reports of more genes contributing to DS, including *SCN2A*, *SCN8A*, and *SCN1B*, reflecting the important contribution of other VGSC subtypes to this disorder [98].

Prior research has predominantly used mouse models to investigate the electrophysiological alterations that occur in Dravet syndrome. These studies consistently highlight an imbalance between electrical excitability and inhibition [104]. Similar results were shown in zebrafish studies, where a dominant loss-of-function mutation in the zebrafish Nav 1.1 (*scn1lab*) impairs its ability to promote neuronal inhibition, whereas a gain-of-function mutation causes overactivation of Nav 1.6 [105]. Nav 1.1 is co-expressed with Nav 1.6 to modulate neuronal inhibition and excitation, helping explain why their imbalance leads to the severe phenotypes seen in DS. Therapeutic approaches seeking to restore this balance seem promising [106]. Selective activation of Nav 1.1 has been explored in a DS mouse model using the venom peptide protein Hm1a [107]. This protein, when administered via intracerebroventricular infusion, restored the function of inhibitory interneurons without altering the function of excitatory neurons [107]. The authors believe that this was possible because Hm1a protein selectively interacts with the inactivation domains of the Nav 1.1 protein and not with other Navs, particularly not with those mediating excitability in excitatory neurons [107]. However, a previous study reported that Hm1a alters the function of Nav 1.2, which is mostly expressed in excitatory neurons [107]. The model system used was Xenopus oocytes, which have no native β subunit expression, which is required for the normal function of the alpha subunits and, therefore, might cause this difference in results [107]. Since zebrafish have native β subunits, it could be a useful model to test this divergent effect of Hm1a.

Colasante et al. [108] explored the potential of using catalytically dead Cas9 (dCas9)-mediated *SCN1A* gene activation to rescue *SCN1A* haploinsufficiency in a mouse model of DS. They screened single-guide RNAs (sgRNAs) to find any capable of stimulating *SCN1A* transcription alongside a dCas9 activation system using P19 cell lines and primary neurons [108]. This approach was then tested in a DS mouse model using Adeno-associated virus (AAV) as the delivery vehicle [108]. Parvalbumin interneurons (a type of inhibitory neurons) in this model recovered their ability to fire action potentials, and febrile seizures were significantly reduced [108], indicating that this could be a useful therapeutic approach for DS and other diseases caused by haploinsufficiency [108]. Similar screening approaches could be explored in the zebrafish model.

There have been reports of significant seizure reduction in some patients with DS using the drug fenfluramine (FA), previously under the trade name “fen-phen,” which acts by increasing serotonin (5-hydroxytryptamine levels 5-HT) levels in the brain (neocortex and hippocampus) [109,110]. Studies in mice and zebrafish showed these effects, and there has been FDA approval of this drug as an adjunctive therapy for DS, Lennox–Gastuat, and sunflower syndrome (severe forms of epilepsy) due to its anticonvulsant proprieties at low doses [111,112,113]. A dual mechanism of action has been proposed for fenfluramine. First, it activates different serotonin (5-hydroxytryptamine) receptor subtypes through binding interactions by its metabolites (D- and L-norfenfluramine), and/or it inhibits the action of the serotonin transporter (SERT), thereby preventing the reuptake of serotonin and increasing its availability in the extracellular space. Increased serotonin levels increase GABA signaling [112]. A second proposed mechanism is through interactions with the sigma-1 receptors: ligand-operated chaperon proteins that modulate both voltage-gated and ligand-gated ion channels, which may combine with effects on neurotransmitters like serotonin [112]. Sigma-1 receptors are activated in response to physiological stressors, such as an imbalance between neuronal excitability and inhibition [114]. Sigma-1 receptors primarily interact with Nav 1.5 channels, which are expressed in both the brain and the heart, and their activity decreases glutamate signaling [112,114,115]. In a study utilizing the *scn1lab* zebrafish model, administration of fenfluramine alongside sigma-1 receptor agonist diminished the drug’s antiseizure activity [109], prompting the need to explore the effects of sigma-1 receptor agonists and antagonists to better regulate neuronal excitability [112]. Although they are localized in the endoplasmic reticulum (ER), sigma-1 receptors can translocate to the plasma membrane and nuclear envelope [116,117]. In addition, sigma-1 receptors have no sequence homology to other mammalian proteins, making them great druggable targets [118].

Besides fenfluramine, other serotonergic system regulators, like clemizole, locaserin, and trazodone, could be effective therapeutics targeting drug-resistant seizures [112]. Despite fenfluramine’s use as an adjunctive therapy for DS, there are concerns about cardiovascular side effects, which was a major setback that led to its withdrawal [110]. Fenfluramine is reported to exert its effects by acting as an agonist of 5-HT-1A, 1D, 2A, 2C, and 3C and an antagonist of 5-HT6 [111]. There are conflicting findings regarding the contributions of other 5HT receptors in fenfluramine’s mechanism of action. Of particular interest, 5-HT2B activation is suggested to be responsible for the cardiovascular side effects. This has been supported with evidence showing that activation of 5-HT2B is linked to heart valve tissue fibrosis [112,119]. Griffin and colleagues reported significant seizure reduction in the *scn1lab* zebrafish model using 5-HT2B agonist, but other studies in rodents and even another *scn1lab* zebrafish model found no inhibition of spontaneous seizures [91]. These inconsistencies make it challenging to determine the precise contributions of the different 5-HT receptors in seizure modulation. More research is needed to better understand fenfluramine’s interactions with the serotonergic receptors and optimize treatment concentration to minimize side effects and increase its applicability across different epilepsy subtypes. Cardiovascular toxicity could also be addressed in the zebrafish model. Zebrafish have homologues of all 14 5HT receptor subtypes and sigma-1 receptors, making them valuable models to study serotonin modulation as a therapeutic target [120].

There are currently seven zebrafish models of DS, based on the zebrafish paralog genes *scn1laa* and *scn1lab*, which are homologs of the human *SCN1A* gene [96], as well as the *SCN2A*, *SCN3A*, and *SCN9A* genes. Most studies report that a homozygous knockout of one of these zebrafish genes mimics the heterozygous *SCN1A* DS phenotype in humans [96]. Since the other paralog is present, a knockout of one is believed to be compensated for by the other. Weuring, Hoekman, Braun, and Koeleman [96] argue that the two paralogs do not fully resemble each other and contain some functionally important regions that are not conserved, such as the S4 voltage sensor domain and the S2–S3 cytoplasmic linker (inactivation gate) in DIV. The lack of conservation in the S4 region of the *SCN1A*, *scn1Lab*, and *scn1Laa* genes suggests that the region responsible for protein interactions differs, potentially leading to variations in how *SCN1B* binds with the human *SCN1A* gene compared to its zebrafish counterparts [96]. This divergence may indicate differing binding interactions or functional roles in the zebrafish versus humans or across species [96]. In addition to the structural differences, the phenotypes resulting from the homozygous knockout of *scn1Laa* or *scn1Lab* using CRISPR-Cas9 differ slightly, with the former showing no locomotor hyperactivity, a unique phenotype reported in most DS models [96]. Given that *scn1Laa* and *scn1Lab* differ substantially, Weuring, Hoekman, Braun, and Koeleman [96] propose that mammalian models will be better alternatives to screen for drugs whose targets are at the molecular level. Additional studies investigating double knockouts of these paralogs could provide deeper insights into their functional differences.

#### 6.1.2. Genetic Epilepsy with Febrile Seizures Plus (GEFS+)

Genetic epilepsy with febrile seizures plus (GEFS+) is an intractable childhood epilepsy characterized by the regular occurrence of febrile seizures at an early stage of childhood growth followed by the development of afebrile seizures later in life [121,122]. It is often characterized by milder epileptic symptoms and commonly diagnosed in families with *SCN1A* and *SCN1B* gain-of-function missense mutations with varying severities [122,123]. These mutations affect the pore-forming domain of the sodium VGSC *SCN1A* and the extracellular Ig-like domain of the β1 subunit [86]. Some studies also show that mutations in *SCN2A*, *SCN3A*, and *SCN9A*, and in some other genes, like *HCN*, *GABRG2*, and *STX1B*, may also lead to GEFS+ [124]. There are numerous missense mutations in the *SCN1A* gene implicated in GEFS+, and the most studied is (R1648H) [122]. This mutation was first found in a family with GEFS+ and results in an amino acid change from arginine (R) to histidine (H) at position 1648 in the S4 segment in DIV of the alpha subunit of the sodium channel Nav 1.1 [84,122]. Several studies in different model systems, ranging from Drosophila to rats and mice, have shown that the R1648H mutation leads to a reduction in inhibitory interneuron excitability [122,125]. These results were similar to studies conducted in a Drosophila K1270T knock-in model, which showed that the mutation causes heat-induced seizure activity due to a temperature-dependent decrease in GABAergic neuron excitability [97].

The exact mechanisms underlying how elevated temperatures promote seizures in the developing brain have not been fully elucidated [126]. In rats and mice, a major technical challenge is generating an optimized method that best mimics elevated body temperatures in humans, which is not lethal and can be monitored as the organism develops [126]. There exists a hyperthermia-induced zebrafish seizure model, which is rapid, reversible, and non-lethal [126]. The generation of this model involves exposing 3–7 dpf larvae to bath-controlled temperatures and recording acute electrographic seizures. The acute electrographic seizures that result in this model show age dependence, strain independence, and no lethality [126]. Using TRPV4 (transient receptor potential vanilloid) channel antagonists, seizures were prevented, suggesting that TRPV4 activation contributes to seizure generation [126]. Similarly, using NMDA (N-methyl-d-aspartate) receptor antagonists, seizures were also prevented, supporting the idea that the glutamatergic system contributes to seizure generation [126]. However, GABA reuptake inhibitors had no effect on seizures. Through gene expression analysis, TRPV4 and NMDA receptors were found to be expressed at different development stages of zebrafish larvae, highlighting the functional importance of these channels [126]. This method can be used to evaluate further receptor agonists and measure their effect on seizure responses. In addition, mutations in any of the genes implicated in GEFS+ could be evaluated to assess their contributions to seizures induced by elevated temperatures in the zebrafish model.

Due to the febrile nature of GEFS+, the contribution of inflammatory cytokines has also been investigated. Clinical data have shown that elevated levels of IL-6 and TNFalpha are associated with a higher possibility of seizure reoccurrence [127]. According to a study conducted by Ling, Wang, Jiang, and Yuan [127], intranasal administration of IL-6 in GEFS+ mice heightened seizure severity, while inhibition of the STAT3-IL-6 pathway using Stattic (a STAT3 phosphorylation inhibitor), and, in a separate study, administration of anti-IL-6 monoclonal antibody, significantly suppressed seizure activity [127]. Together, these illustrate the functional contribution of inflammatory cytokines such as the STAT3-IL6 pathway to the pathogenesis and severity of GEFS+ [127]. This suggests that immunosuppressive therapy targeting the STAT3-IL-6 pathway could offer potential therapeutic opportunities [127]. Besides IL6/STAT3, other critical inflammatory cytokines could be explored. Previous studies have shown that seizures activate glial and non-neuronal cells, leading to increased expression of IL-6, TNFalpha, and interferons, which intensify epileptic activity. Although not reported in GEFS+, it has been shown in a DS *Scn1lab* model of epilepsy that epileptic seizure promotes neuroinflammation in the brain through the activation of microglia cells [128]. Interestingly, however, these upregulated levels of microglia offer a neuroprotective role [128]. This was demonstrated by comparing microglia depleted in a DS *Scn1lab* model, which showed an increase in epileptiform activity with that of the DS *Scn1lab* model with intact microglia [128]. Thus, microglia therapy could be explored in a broader scope to assess the therapeutic potential.

### 6.2. SCN2A

The human *SCN2A* gene encodes the VGSC protein Nav 1.2. Nav 1.2 is one of the four Nav channels expressed throughout the CNS, in addition to Nav 1.1, Nav 1.3, and Nav 1.6 [129]. Together, they make up the most abundant sodium channel subtypes in the brain and are responsible for most of the known sodium channelopathies in the brain [35]. Nav 1.2 resides in the cell membrane and binds to ankyrin G, which anchors it to the membrane and promotes its interaction with calmodulin [130]. Mutations in the ankyrin-binding motifs hinder this binding interaction, with knockout studies of ankyrin G leading to reduction of Nav 1.2 expression in the axon initial segment (AIS, a unique compartment where the axon emerges from the cell body, made up of a protein complex that drives neuronal excitability and polarity) [131]. Ankyrin G plays a critical role in the distribution of Nav 1.2 to the AIS and the nodes of Ranvier [132]. Nav 1.2 also plays an important role in the backpropagation of action potentials into the soma and dendrites, which are essential for synchrony and synaptic plasticity (changes in neuronal connections essential for learning and memory) [133]. More than 150 mutations have been identified within the *SCN2A* gene, which are implicated in autism spectrum disorders, intellectual disabilities, benign familial neonatal infantile seizures, and developmental and epileptic encephalopathy [94,134]. In individuals with either autism spectrum disorders or intellectual disability, the mutations tend to result in a loss of function of the gene, whereas in early-onset infantile epilepsies, mutations tend to be gain of function [94]. There are exceptions to this in the case of late-onset infantile epilepsies, which are due to loss-of-function mutations [94,106,135].

#### Early- and Late-Onset Infantile Epilepsies

Sodium channel blockers are effective in early-onset epilepsies but typically not in late-onset epilepsies [57]. Early-onset infantile epilepsies often tend to be self-remitting, with onset occurring from a few days to weeks after birth and lasting until about two years of age [35]. This self-remission is believed to occur through several mechanisms, which primarily involve the developmental transition of sodium channel expression and function [35]. Nav 1.2 is highly expressed in excitatory neurons, specifically in the axon initial segments during gestation [132]. It is also referred to as the primary nodal channel during early development. As development progresses, the Nav 1.6 channel gradually replaces Nav 1.2, becoming the critical channel for action potential initiation and propagation in that region [136]. The presence of these sodium channels in this region mediates saltatory conduction in myelinated neurons. In adult unmyelinated neurons, however, Nav 1.2 continues to be expressed along the entire length of the axon. Although Nav 1.6 is more commonly associated with saltatory conduction in myelinated neurons, it can also support continuous conduction in unmyelinated neurons [137,138]. Thus, overexpression of Nav 1.6 in these regions is believed to partially compensate for functional deficits caused by Nav 1.2 mutations [132]. This observation aligns with the age-dependent resolution of benign familial neonatal–infantile seizures (BFNISs), which are hypothesized to result from the physiological reorganization of axon initial segments during development. Nav 1.6 overexpression studies and the overexpression effect on early-onset epilepsies could be explored in further detail [139].

Another proposed mechanism involves alternative splicing of Nav 1.2, which produces neonatal (Nav 1.2N) and adult (Nav 1.2A) isoforms. Nav 1.2N is predominantly expressed during early development and exhibits lower excitability compared to the adult Nav 1.2 [129]. Although the functional importance of this is not clearly established, some studies hypothesize that the lower excitability in the Nav 1.2N isoform renders a protective mechanism against excessive neuronal firing during gestation or early life [129]. This hypothesis is supported by a study reporting that the mutated neonatal isoforms mimicking the channel properties of the adult isoform with a lower threshold of excitation (i.e., more excitable) are detrimental to the developing brain [35].

A study examining the effects of five common Nav 1.2 mutations in both neonatal and adult isoforms in a HEK-derived cell line found that the three out of the five (T236S, E999K, S1336Y) mutations produced a larger effect (significantly depolarized voltage dependence) on the neonatal isoforms than on the adult isoforms [140]. Mutually exclusive splicing (a form of pre-mRNA processing) occurs in about five of the sodium channels (*SCN1A*, *SCN2A*, *SCN5A*, *SCN8A*, *SCN9A*), where only one of the two forms of exons are incorporated into the final mRNA transcript to produce neonatal and adult isoforms [140]. In the case of Nav 1.2, this splicing event leads to the developmentally regulated insertion of exon 5, where exon 5N is incorporated into the neonatal isoform and exon 5A into the adult isoform [129]. These exons differ only by a single amino acid at position 209 (N209D), where the neonatal isoform contains asparagine (N) and the adult isoform has aspartic acid (D) [129]. This switch influences four key aspects of channel conductance: activation, conductance, inactivation, and recovery from inactivation [129]. Similarly, a change in the aspartate residue in the neonatal Nav 1.5 isoform to lysine in the adult Nav 1.5 isoform results in an increase in the charge and sodium influx of the neonatal channel. Nav 1.1 5N channels also recover more quickly from fast inactivation than the adult isoform [129]. The Nav 1.1 neonatal isoform shows a more sensitive response to intracellular fluoride ions and changing temperatures than the adult isoform [141]. Nav 1.6 isoforms exhibit tissue-specific expression, with the adult transcript mostly expressed in the brain having an intact exon 18 (without an in-frame stop codon), and the neonatal isoform expressed in other tissues besides the brain having a truncated protein due to the presence of an in-frame stop codon in exon 18 [142]. Generally, the switch between the neonatal and adult isoforms leads to an altered structure between the short linker of the S3 transmembrane segment and the voltage sensor (S4) in domain I [140] Given the potential differential contributions of neonatal and adult isoforms of the sodium channels, more research is needed to understand whether there are appropriate windows of treatment. The zebrafish model, with transparent embryos and rapid development [143], may be particularly suited for assessing the impact of isoform switching in greater detail during early development.

### 6.3. SCN3A

*SCN3A* encodes Nav 1.3, which is highly expressed in the embryonic brain and involved in the generation and propagation of action potentials in excitable cells [144]. It has also been found to be highly expressed in the basal/outer radial glia and migratory newborn neurons, suggesting an additional non-action potential role during early development [145]. Its expression peaks at birth and then declines after the second postnatal week to very low levels in adulthood [144]. Nav 1.3 expression, however, can be upregulated under pathological conditions such as neural injury or recently reported epilepsy [146]. Nav 1.3 channels possess intrinsic properties such as rapid recovery from inactivation and the ability to sustain high-frequency firing [147], suggesting a potential contribution to neuronal hyperexcitability when abnormally expressed. Many studies report the involvement of this channel in neuropathic pain, inspired by findings that identify it as the only Nav 1.3 transcript upregulated after nerve injury in rat dorsal root ganglia (DRG) [146]. Its involvement in epilepsy is supported by studies that have shown that the *SCN3A* mRNA is highly expressed in CA4 hilar cells within the epileptic hippocampus [147]. Currently, there are thirteen reported variants of *SCN3A* implicated in epilepsy [146,147,148]. While some *SCN3A* variants have been linked to milder phenotypes, such as focal epilepsy and generalized epilepsy with febrile seizures plus (GEFS+), there are severe forms, such as early infantile epileptic encephalopathy (EIEE) [149]. Affected patients may also have characteristic structural abnormalities, such as polymicrogyria (where there is excessive folding of the cerebral cortex), global development delay, and speech and oral motor dysfunction in the absence of epilepsy [144,145,147].

Both gain-of-function and loss-of-function mutations have been identified in *SCN3A* [150]. In cases of loss of function, the presentations are milder, like the cases of focal epilepsies and GEFS+, and are characterized by depolarizing shifts in voltage-dependent activation and inactivation, as well as slow recovery from inactivation, which indicates a reduction in channel activity [150]. Heterozygous missense mutations in *SCN3A* have been identified as a cause of early infantile epileptic encephalopathy (EIEE), with three causative variants reported in four patients [149]. These mutations affect highly conserved residues and result in gain-of-function effects such as activation at hyperpolarizing potentials and increased persistent sodium current [146]. The gain-of-function effects observed may be partially repressed by ASMs such as lacosamide, phenytoin, and valproic acid [144]. While both humans and mice show high *SCN3A* expression during early development followed by a decline into adulthood, this decline is more pronounced in mice [150]. Therefore, a limitation of using mouse models to study *SCN3A*-related conditions is that the adult expression pattern in mice may not accurately reflect that in humans [150]. In addition, Cummins et al. [151] reported possible methodological limitations. Studies on the expression of Nav 1.3 in mammalian cells and spinal sensory neurons showed differences in association with β subunits, which may alter the function of the α subunit [151]. Currently, there are no reports of *SCN3A* mutation studies in zebrafish. However, given the phylogenetic similarity between the mammalian *SCN3A* and the two zebrafish genes *scn1laa* and *scn1lab* [87], *SCN3A* variants could potentially be modeled in zebrafish to screen for antiseizure drugs and further assess the impact of modulatory or accessory proteins such as β subunits on the channel function.

### 6.4. SCN8A

*SCN8A* encodes the alpha subunit of the sodium channel Nav 1.6, which is predominantly found in neurons in the CNS and PNS, with minimal expression in cardiac tissues [152]. It is concentrated in the AIS and the nodes of Ranvier, which are the sites of action potential initiation and regeneration, respectively, during the saltatory transmission [153]. Nav 1.6 interacts with the microtubule-binding protein (MAP1B) through its cytoplasmic NT region, which stabilizes it to the distal AIS, preventing rapid endocytosis [35]. While Nav 1.2 is involved in backpropagation at the AIS, Nav 1.6 generates forward propagation [35]. Earlier *SCN8A* mutations studies reported that most of the variants occurred in the transmembrane segment, the inactivation gate, and the cytoplasmic CT domain [154]. Recent work has shown that there are some variants found in the NT of mouse Nav 1.6 that prevent the correct localization of the channel, which causes Nav 1.6 retention in the Golgi apparatus [35]. There are more than 300 individuals with reported *SCN8A* epileptic encephalopathies [154]. Loss-of-function mutations in *SCN8A* are generally less severe, clinically presenting as myoclonus and isolated intellectual disability [152]. On the other hand, gain-of-function mutations lead to overall neuronal hyperexcitability and epileptic seizures due to premature channel opening, impaired inactivation, and elevated persistent currents [152].

The first *SCN8A* variant identified in an affected child was N1768D [154]. Studies of this variant in transfected neurons showed a large increase in persistent current and elevated firing [154]. Using TALEN endonuclease, this variant was knocked into exon 26 of mouse *Scn8a*, and spontaneous seizures were observed in heterozygous adults at 2 months of age [154]. Using the same genetic approach, a conditional expression of the R1872W mutation was generated [154]. Activation of the mutant channel at different developmental stages allowed for the characterization of the effect of the mutation at different time points, as well as assessment of the effects on excitatory verses inhibitory neurons [154]. Global activation of the mutant channel early in development led to sudden seizure onset at 14 days of age, followed by death within 24 h after the first observed seizure. Activation of R1872W in the forebrain was sufficient to trigger seizures and lethality, while expression in inhibitory neurons produced no pathogenic outcome [154]. Expression of the mutant channel later in development or in adult mice resulted in spontaneous seizures and sudden death [154], showing that the adult neurons were highly susceptible to the effects of the mutant channel. Although Nav 1.6 expression is minimal in cardiomyocytes, its presence is sufficient to induce cardiac arrythmias in N1738D mice, resulting in sudden death or lethality [154]. Dravet syndrome (DS) was also shown to increase the functional Nav 1.6 expression in transverse tubule nanodomains of cardiomyocytes, promoting calcium-mediated cardiac arrythmias responsible for SUDEP [155]. Notably, a cardiac-specific reduction in Nav 1.6 was shown to decrease the arrythmia burden and improve survival in DS mice [155]. These findings suggest that the maladaptive remodeling of Nav 1.6 in the heart contributes to mortality in adult DS mice. Targeting cardiac Nav 1.6 could therefore serve as a potential therapeutic approach for preventing SUDEP in DS and mitigating the lethality associated with Nav 1.6 mutations [155].

To the best of our knowledge, there are no established zebrafish models of *SCN8A* gain-of-function mutations. However, *SCN8A* knockouts were studied to assess the functional consequences of its loss [90]. Movement disorders are commonly observed in *SCN8A*-related phenotypes, and the zebrafish model has demonstrated that *SCN8A* expression is critical for normal motility during early embryonic development [90]. The zebrafish *scn8aa* gene is specifically expressed in Rohon–Beard (RH) neurons and trigeminal ganglia as early as 16–17 hpf [90]. Increased *scn8aa* channel expression leads to an increase in RH neuronal currents, whereas *scn8aa* knockout leads to impaired development of secondary motor neurons, reduced spontaneous contractions, and reduced tactile sensitivity [90].

## 7. Conclusion: VGSCs as Therapeutic Targets

Sodium channels are druggable targets, and small molecules can be engineered to interact with them, increasing or decreasing neuronal excitability. The alpha subunit of VGSCs is the target for many ASMs because it forms the pore domain of the channels. VGSCs harbor the most identified and studied channelopathy mutations. The first generation of ASMs were often pore-blocking drugs, which were employed for treatment of channelopathies like epilepsy. However, their clinical utility is often limited by non-specific binding interactions, leading to side effects such as life-threatening arrhythmias. On a brighter note, recent advancements have led to the development of alternative targeting strategies, such as subtype-specific inhibitors and drugs that target specific voltage sensors (integral domains in VGSCs, primarily involved in ion sensing). Currently, serotonergic drugs are being investigated as promising candidates to address drug-resistant epilepsies. Other novel therapeutic approaches include peptide-based CNS therapeutics. Although a major limitation is their inability to cross the blood–brain barrier, computational methods have been developed to standardize the scoring of genetic variants found in epilepsy patients, which could improve personalized treatment strategies. Since different model systems yield slightly varying results, and even within the same model system there exist subtle differences in treatment outcomes, a broader and more integrative approach is needed.

## 8. Future Directions

Future studies could aim to map out therapeutic targets, explore combination treatments, and assess their interactions. In addition, gene-editing approaches such as the use of CRISPR-Cas9 can be employed. Since some of the mutations tend to be embryonic lethal, conditional models using the Cre-lox system may also be explored. Patient-derived models offer more direct insights into the effects of specific mutations and therefore could offer more consistent outcomes and reduce the variability associated with the different model systems discussed above.

## Figures and Tables

**Table 1:** Zebrafish vs. rodents as model organisms for VGSC research.

Characteristics	Zebrafish Models	Rodent Models
Genetic homology	High sequence homology with human disease genes (84%) [4,56]	Overall high genetic and physiological homology with humans (~90%) [72]
Sodium channel isoforms	Different sodium channel isoforms, e.g., zebrafish express *scn1lab*, an ortholog to *SCN1A* in humans [73]	Structure and function of VGSCs conserved in mammals
Nervous system complexity	Primitive nervous system; limited ability to simulate complex neuronal diseases	Homologous brain networks with primates [62]
Electrophysiology	Patch-clamp is challenging due to small cell size, but protocols like in vivo whole-cell and multichannel recordings are established [74,75]	In vivo patch-clamp methods are established but often require anesthesia and invasive electrode insertion, raising ethical concerns [76]
Genetic manipulation	External fertilization allows easy genetic manipulation and access to one-cell zygotes for injections [64]	In vitro, ex vitro, and in vivo experimental models, but more resource-intensive
Behavioral studies	While many behavior analyses have been established for zebrafish, translating results to human models can be challenging	Rodent models can capture behavioral components that can be translated into human neurological disorders and psychiatry [77]
Development	High proliferation, oviparity, and translucence of developing embryos make observation of early development stages easier	In utero development, prolonged growth, and maternal dependence complicate early embryogenesis studies and toxin exposure assessments
Ethical considerations	Fewer ethical concerns of embryos and larvae up to 5 dpf [70,71]	Ethical concerns regarding invasive procedures [63]
Cost of maintenance	Lower cost of maintenance	Higher cost of maintenance [63]

**Table 2:** Human SCNA genes and their corresponding zebrafish orthologs.

Human Gene	Encoded Protein	Zebrafish Orthologs	Expression Pattern	Identity to Homolog	References
*SCN1A*	Nav 1.1	*scn1laa*/*scn1lab*	Central nervous system (throughout the brain, eye, ventral regions of spinal cord)	77%	[87]
*SCN2A*	Nav 1.2				
*SCN3A*	Nav 1.3				
*SCN9A*	Nav 1.7				
*SCN4A*	Nav 1.4	*scn4aa*/*scn4ab*	Skeletal muscle/mesodermal tissues	NA	[87]
*SCN5A*	Nav 1.5	*scn5Laa*/*scn5Lab*	Cardiac tissues	60–65%	[87,89]
*SCN10A*	Nav 1.8				
*SCN11A*	Nav 1.9				
*SCN8A*	Nav 1.6	*scn8aa*/*scn8ab*	Rohon–Beard neurons and trigeminal ganglion (16–17 hpf)	83%	[87,90,91]
